# Mistakes of the Types

**Published:** 1863-01

**Authors:** 


### MISTAKES OF THE TYPES.

THE types made terrible havoc of our copy in the last issue of the "Register." It turned some of our remarks into second-rate nonsense; but as they would not have been very wise if printed correctly, we will try and not complain. We did n't see the proof." W.

